# A novel role of master regulator transcription factor in anti-tumor immunity and cancer immunotherapy

**DOI:** 10.7150/thno.83486

**Published:** 2023-03-16

**Authors:** Uttam K. Sinha, De-Chen Lin

**Affiliations:** 1 Department of Otolaryngology, Keck School of Medicine, University of Southern California, Los Angeles, USA.; 2 Center for Craniofacial Molecular Biology, Herman Ostrow School of Dentistry, Norris Comprehensive Cancer Center, University of Southern California, Los Angeles, USA.

## Abstract

Gene expression network in cancer cells is orchestrated by a small number of master regulator transcription factors (MRTFs), which play a prominent role in regulating cancer intrinsic hallmarks, such as sustaining proliferative signaling, evading growth suppressors, resisting cell death, etc. A new study reports a new role of one MRTF, KLF5, in regulating tumor microenvironment in an extrinsic manner. These findings not only reveal novel mechanistic underpinnings of tumor evasion from immune destruction but also broaden our understanding of the transcriptional deregulation in cancer biology.

Although nearly half of ~1,000 human transcription factor (TFs) can be detected in any given cell type, only a small number are critical for establishing cell identity by controlling specific gene expression programs^1^,^2^. They are often referred to as master regulator TFs (MRTFs), which co-occupy most cell-type-specific (super)enhancers^3^-^5^ and orchestrate the gene regulatory network for cell-type-specific functions. Considering that transcriptional dysregulation is a hallmark of cancer^6^, characterization of MRTFs has imperative significance for the understanding of gene expression networks in cancer. Indeed, in several tumor types, investigations of MRTF-mediated programs have successfully deciphered cancer-specific pathways, uncovered tumor dependencies, and implicated neoplastic cell-of-origins. For example, MRTF characterization led to the discovery of three subtypes of neuroblastomas, each driven by distinct sets of MRTFs^7^. Similarly, studies of MRTFs in medulloblastoma^8^ and ependymoma^9^ revealed prominent subtype-specific cellular origins and tumor addictions.

Nevertheless, most of prior work on cancer MRTFs have been focused on their tumor-intrinsic functions. Whether and how MRTFs regulate cancer biology in a non-cancer cell autonomous manner has not been extensively explored. In the published paper of *Theranostics*, Wu *et al.* address this critical question by studying Krüppel-like Factor 5 (KLF5), a quintessential MRTF^10^. Indeed, KLF5 has been characterized as a key MRTF across multiple cancer types, including breast, esophageal, colorectal and pancreatic cancers^11^-^15^.

Using breast and colon cancer syngeneic murine models, the authors first noted a marked increase of infiltration of CD8^+^ T cells into tumors with Klf5 knockout or knockdown compared with the control group, as measured by the immunohistochemistry staining. On the other hand, tumors with Klf5 overexpression had reduced CD8^+^ T cell infiltration. These intra-tumoral CD8^+^ T were functional, since their depletion by an anti-CD8 neutralizing antibody facilitated the xenograft growth. To understand how Klf5 regulates the tumor-immune microenvironment (TME), RNA-sequencing analysis was performed and revealed that Klf5-knockdown induced a prominent enrichment of T-cell proliferation, differentiation, chemotaxis and activation, among other immune pathways. Deconvolutional analysis showed that naïve and activated dendritic cells (DCs), NK cells, CD4^+^ effector memory T cells and CD8^+^ effector memory T cells were enriched within tumors with Klf5 knockdown. Moreover, opposite changes in the TME composition were observed in Klf5-overexpressing tumors, suggesting that tumor-intrinsic Klf5 expression modulates immune compositions in the TME by regulating the biology of T cells.

Further probing the underlying mechanism, the authors placed their attention on their prior working showing that KLF5 promoted PGE2 production in breast cancer^16^, because PGE2 is known to have immune-regulating function. Here, using ChIP, dual luciferase assay and mutagenesis approach, they identified a novel Klf5/Cox2 axis enhancing the production of PGE2. Importantly, the activation of the Klf5/Cox2 axis reduced the number of tumor-infiltrating CD8^+^T cells, and Cox2 inhibition by a small-molecule inhibitor (CEL) partially increased CD8^+^T cell infiltration. Moreover, multicolor flow cytometry revealed that Klf5 silencing led to augmentation of subpopulations of proliferating T cells (Icos+), cytotoxic T cells (Ifnγ^+^) as well asl Pd1-positive T cells. Therefore, blocking the Klf5/Cox2 pathway in cancer cells enhances the quantity and functionality of anti-tumor T cells.

Prompted by these observations, the authors next conducted a preclinical testing to investigate whether inhibition of the Klf5/Cox2 axis reinforces the efficiency of immune checkpoint blockade therapy. Notably, while mice with EMT6 or CT26 tumors (expressing high Klf5 endogenously) were unresponsive to anti-Pd1 monotherapy, the anti-Pd1 blocker resulted in clear tumor regression and prolonged survival in mice with Klf5 knockdown tumors. Moreover, combinatorial treatment using the anti-Pd1 antibody and inhibitors against the Klf5/Cox2 axis potently reduced tumor growth, leading to tumor eradication and increased overall survival.

Extending these murine data to human cancers, the authors reanalyzed public single-cell RNA-seq and spatial transcriptomic data on breast cancer patients^17^,^18^. Importantly, compared with KLF5^high^ tumors, the number of CD4^+^ and CD8^+^ T cells was higher in samples from KLF5^low^ tumors. The fractions of CD4^+^IFNγ^+^ Tem, CD8^+^GZMB^+^ Tem and CD8^+^CXCR6^+^ Trm cells were also upregulated in KLF5^low^ patient tumors. Finally, to assess the prognostic value of KLF5/COX2-driven immune profiles, the authors generated a KLF5/COX2-immune score based on their transcriptome data. Notably, in both breast and colon cancer patient cohorts, the KLF5/COX2-immune score is significantly associated with patient survival. Together, these results establish a novel role of KLF5, a key cancer MRTF, in the regulation of antitumor immunity that is independent of its tumor-intrinsic function. While it remains to be seen whether other MRTFs also possess similar tumor-extrinsic functions, this study represents a significant step forward in understanding the transcriptional deregulation in cancer biology while identifying potential new avenues to enhance the efficacy of immunotherapy.
